# Single Procedure of Transcatheter Aortic and Mitral Valve-in-Valve Implantation in a Patient With Cardiogenic Shock

**DOI:** 10.1016/j.jaccas.2025.104354

**Published:** 2025-10-22

**Authors:** Filippo Leidi, Simone Tresoldi, Ivan F. Calchera, Matteo Pozzi, Giuseppe Trocino, Giovanni Marchetto, Maddalena Lettino, Pietro Vandoni, Stefano Righetti

**Affiliations:** a Interventional Cardiology Department, Fondazione IRCCS San Gerardo dei Tintori, Monza, Italy; b School of Medicine and Surgery, University of Milano-Bicocca, Milan, Italy; c Intensive Care Unit, Fondazione IRCCS San Gerardo dei Tintori, Monza, Italy; d Cardiovascular Diagnostics Department, Fondazione IRCCS San Gerardo dei Tintori, Monza, Italy; e Cardiac Surgery Department, Fondazione IRCCS San Gerardo dei Tintori, Monza, Italy; f Cardiothoracic and Vascular Department, Fondazione IRCCS San Gerardo dei Tintori, Monza, Italy

**Keywords:** acute heart failure, aortic valve, mitral valve, pulmonary edema, valve replacement

## Abstract

**Background:**

Transcatheter valve-in-valve (ViV) implantation is the treatment of choice for degenerated surgical bioprostheses, particularly in high-risk surgical patients.

**Case Summary:**

A 50-year-old man with severe dysfunction of a surgical aortic and mitral bioprosthesis rapidly developed cardiogenic shock. A dedicated computed tomography scan for ViV screening was not feasible owing to hemodynamic instability. Preprocedural screening was conducted using information from the ViV app, transesophageal echocardiography, and pulmonary computed tomography performed in the emergency department. A combined aortic and mitral ViV implantation was successfully performed in a single session under sedation. The patient was safely discharged and at 4 years of follow-up had not experienced any cardiovascular event.

**Discussion:**

This case is the first report to our knowledge of a combined aortic and mitral ViV implantation performed under sedation in a patient in cardiogenic shock.

**Take-Home Message:**

In hemodynamically unstable patients, this procedure appears to be safe even without use of general anesthesia.

## Visual Summary

**Figure:**
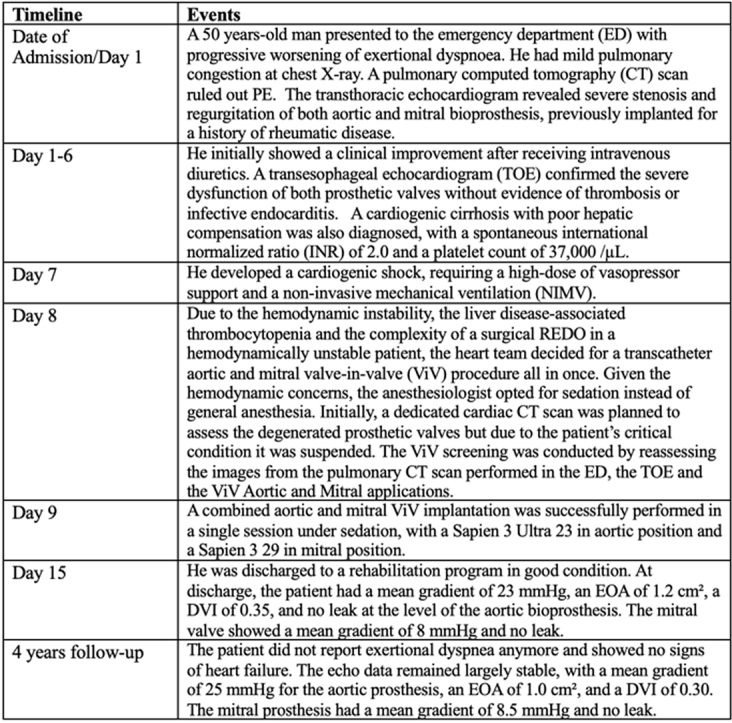

**Equipment List:** 

• EMERALD J-TIP 175 CM (Cordis) • EMERALD STANDARD EXCHANGE J-TIP J-Tip 032 inch × 3 mm × 260 cm (Cordis) • SAFARI2 275 cm XSML CURVE (SGL) (Boston Scientific) • STARTER Guidewire 0.035 × 180 cm (Boston Scientific) • Edwards Commander delivery system for Sapien 23 mm (Edward Lifesciences) • ARMADA 35 14 × 40 mm × 80 cm (Abbott) • SUPER TORQUE 6-FR AL1 (Cordis) • PIGTAIL 6-F × 110 CM SUPER TORQUE PLUS (Cordis) • ESHEATH FOR COMMANDER 23 mm and 26 mm (Edwards Lifesciences) • Edwards Commander delivery system for Sapien 29 mm (Edward Lifesciences) • Introducer sheath 6-F × 10 cm (Terumo) • Introducer sheath 9-F × 11 cm (Cordis) • SL0 sheath and dilator (St Jude Medical) • PROGLIDE CLOSURE SYSTEM (Abbott) × 2 • SAPIEN 3 ULTRA 23 mm (Edwards Lifesciences) • SAPIEN 3 ULTRA 29 mm (Edwards Lifesciences)

Equipment used during the procedure is listed with manufacturers.

## History of Presentation

**Figure 1:**
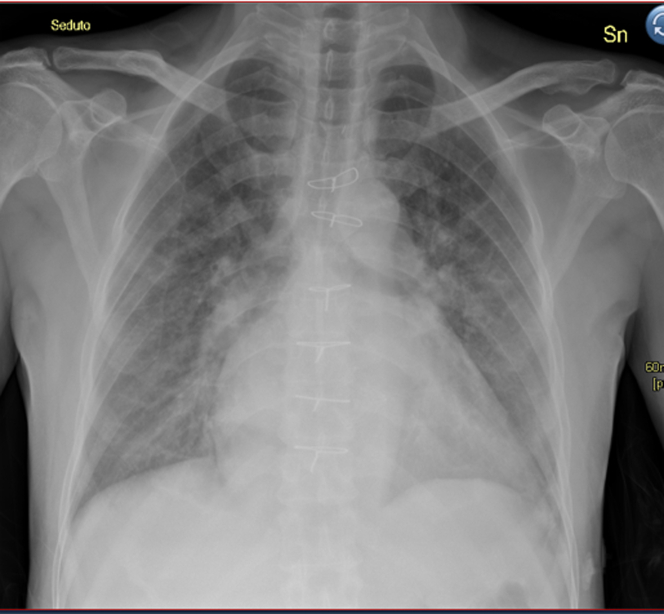
Chest X-Ray at Arrival in Emergency Department At the admission, the chest x-ray showed mild pulmonary congestion.

A 50-year-old man presented to the emergency department (ED) with progressive worsening of exertional dyspnea. On admission, he was hemodynamically stable with an oxygen saturation of 97% on room air. He had mild peripheral and pulmonary congestion (Figure 1) with N-terminal pro–B-type natriuretic peptide of 1,321 pg/mL. Physical examination revealed rhythmic heart sounds with a 4/6 systolic-diastolic murmur.

**Take-Home Messages**

• Combined transcatheter aortic and mitral ViV implantation can be performed with good outcomes even under sedation and in a patient presenting with cardiogenic shock.
• In the absence of a dedicated CT scan, it is possible to perform adequate preprocedural screening by combining the information obtained from aortic and mitral ViV apps with the images from TEE and a pulmonary CT scan.

## Past Medical History

At age 41, the patient developed severe aortic and mitral regurgitation as a result of rheumatic heart disease in childhood. Owing to doubts regarding patient compliance with anticoagulant therapy, at that time he underwent a successful surgical mitral and aortic valve replacement with bioprosthetic valves (Trifecta 23 [Abbott] in the aortic position and Biocor Epic 29 [Abbott] in the mitral position).

## Differential Diagnosis

The differential diagnosis included pulmonary edema owing to valvular dysfunction, pulmonary embolism (PE), and pneumonia.

## Investigations

An urgent pulmonary computed tomography (CT) scan ruled out PE. Transthoracic echocardiography revealed mild left ventricular dilatation without significant hypertrophy, a preserved ejection fraction, and a hypokinetic right ventricle of normal size (Video 1, Video 2). However, the bioprosthetic aortic valve had a stage 3 dysfunction^1^ with both severe stenosis and regurgitation, with a mean gradient of 48 mm Hg, a Doppler velocity index (DVI) of 0.15, and an effective orifice area (EOA) of 0.7 cm^2^ (Video 1). Also, the bioprosthetic mitral valve showed severe stenosis, with a mean gradient of 18 mm Hg, and massive regurgitation. Additionally, severe tricuspid regurgitation was present with a high probability of pulmonary hypertension. Transesophageal echocardiography (TEE) confirmed severe dysfunction of both prosthetic valves without evidence of thrombosis or infective endocarditis (Video 2, Video 3). Coronary angiography excluded coronary lesions. During hospitalization, cardiogenic cirrhosis with poor hepatic compensation was also diagnosed, with a spontaneous international normalized ratio of 2.0 and a platelet count of 37,000/μL and a total bilirubin of 2.9 mg/dL.

## Management

**Figure 2:**
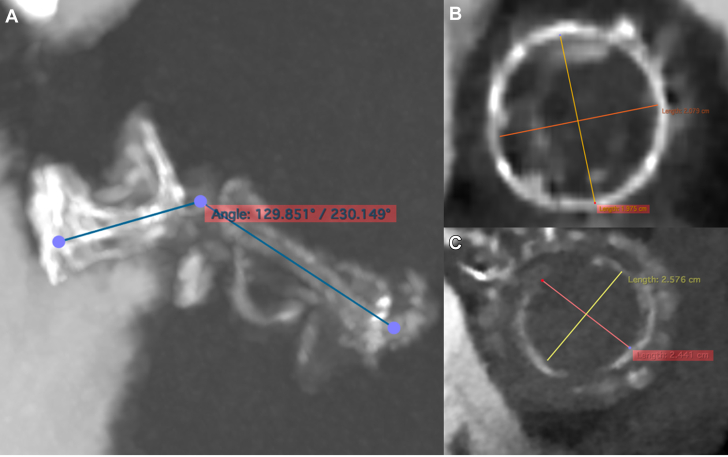
Preprocedural Screening Using Nonsynchronized CT Scan Acquired in the Emergency Department (A) The aorto-mitral angle of 129° reduced the risk of left ventricle outflow tract obstruction. (B) The Trifecta 23 true internal diameter of 21 mm was confirmed on the pulmonary computed tomography (CT) scan. (C) The Biocor Epic 29 true internal diameter of 25 mm was confirmed on the pulmonary CT scan.

The patient initially showed clinical improvement after receiving intravenous diuretics. However, 7 days after admission, he developed cardiogenic shock, requiring a high dose of vasopressor support and noninvasive mechanical ventilation (NIMV). Owing to the patient’s hemodynamic instability, liver disease–associated thrombocytopenia, and the complexity of a redo operation in a hemodynamically unstable patient, the heart team decided to perform a simultaneous transcatheter aortic and mitral valve-in-valve (ViV) procedure. Given the hemodynamic concerns, the anesthesiologist opted for sedation instead of general anesthesia. Initially, a dedicated cardiac CT scan was planned to assess the degenerated prosthetic valves, but it was suspended owing to the patient's critical condition. ViV screening was conducted by reassessing the images from the pulmonary CT scan performed in the ED, TEE, and ViV Aortic^2^ and Mitral digital apps.^3^ The true internal diameter (ID) of 21 mm of the aortic bioprosthesis was determined based on the app and confirmed on the CT scan (Figure 2).

**Figure 3:**
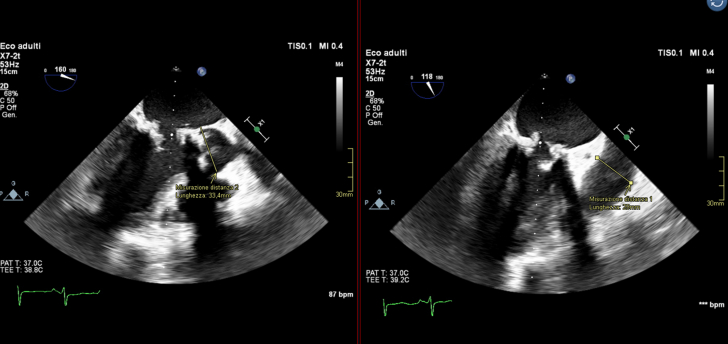
Preprocedural Screening Using TEE, Evaluating the Risk of Left Ventricle Outflow Tract Obstruction The dimensions of the aortic sinuses and the sinotubular junction were 33 mm and 29 mm, respectively, on transesophageal echocardiography (TEE). This reduced the risk of coronary obstruction.

**Figure 4:**
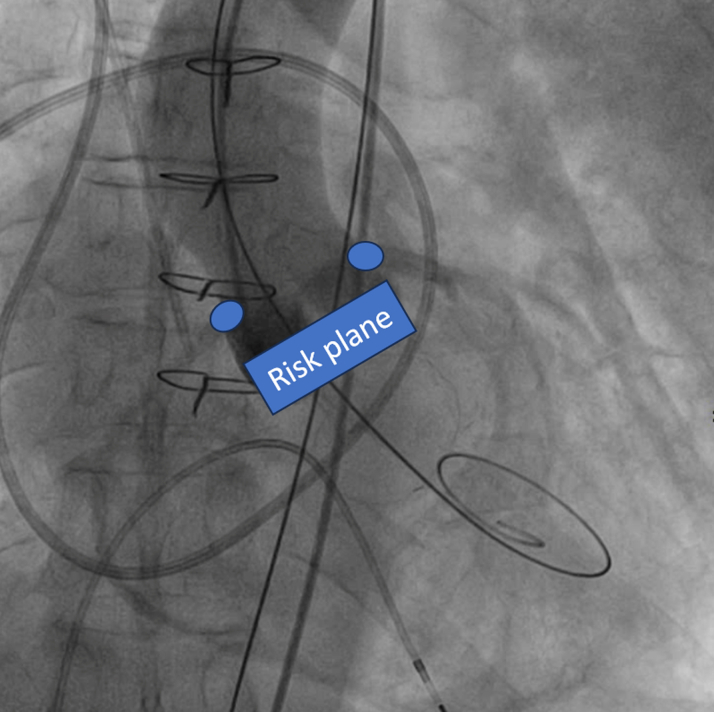
Angiography, Coplanar View, Evaluating the Risk of Coronary Obstruction The posts of the Trifecta 23 were located below the ostia of both coronary arteries.

Owing to the risk of coronary obstruction, the degenerated bioprosthesis was stented, with the pericardial leaflets sutured outside the stent. It was not possible to measure the virtual-to-coronary and virtual-to-sinotubular junction distances, as the CT scan performed in the ED was acquired in the venous phase. As a result, the aortic wall and the coronary ostia could not be clearly identified. However, the Valsalva sinuses were wide on TEE, measuring approximately 33 mm, and the sinotubular junction was 29 mm (Figure 3). Before ViV implantation, aortic angiography was performed with the sewing ring in a coplanar view,^4^ confirming that the origin of both coronary arteries was above the posts of the degenerated surgical valve (Figure 4, Video 4). Ultrasound of both femoral arteries showed no atherosclerotic plaques and diameters >5.5 mm. It was decided to implant a Sapien 3 Ultra 23 [Edwards Lifescience] valve via a transfemoral approach. The mitral bioprosthesis had a true ID of 25 mm, as confirmed on the ED CT scan (Figure 2). Regarding the risk of left ventricular outflow tract (LVOT) obstruction, the prosthesis had the porcine leaflets sutured inside the stent. From the nondedicated CT scan, it was still possible to reconstruct the relationship between the aortic and the mitral bioprostheses, highlighting an aorto-mitral angle of 129° (Figure 2). Additionally, the left ventricle was mildly dilated without excessive hypertrophy.

**Figure 5:**
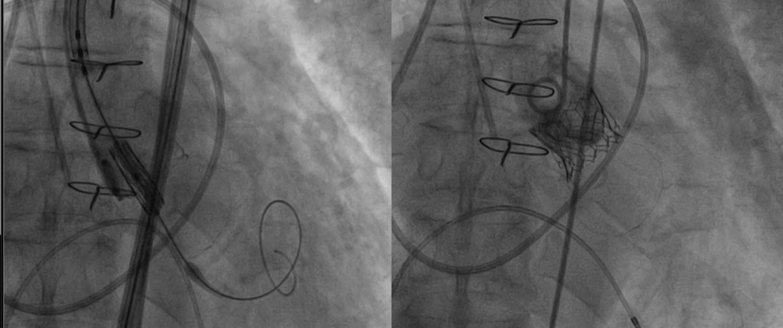
Intraprocedural Angiography, Aortic Valve-in-Valve Implantation The Sapien 3 Ultra 23 valve was implanted in the degenerated Trifecta 23 surgical valve with optimal result.

**Figure 6:**
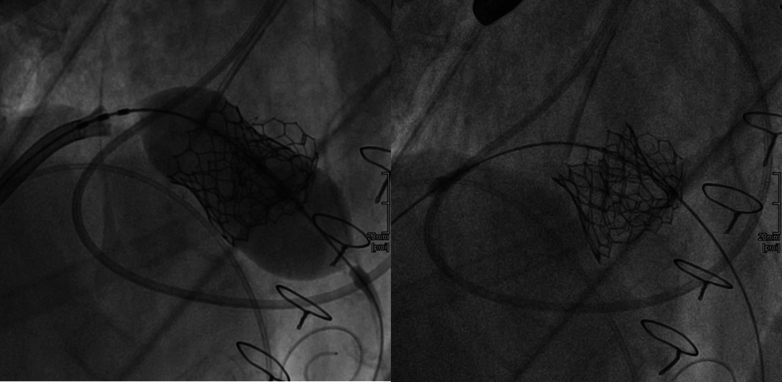
Intraprocedural Angiography, Mitral Valve-in-Valve Implantation The Sapien 3 29 valve was implanted in the degenerated Biocor Epic 29 surgical valve with optimal result.

Owing to the absence of arterial phase images of the CT scan, it was not possible to estimate the predicted neo-LVOT area. However, based on the previous considerations, the risk of LVOT obstruction was estimated as low.^5^ A Sapien 3 29 was chosen for the mitral ViV. The procedure was performed 9 days after admission. The Sapien 3 Ultra 23 valve was implanted in the degenerated Trifecta 23 surgical valve via left femoral access. After advancing a stiff Safari2 Small guidewire [Boston Scientific] into the left ventricle, the valve was deployed under rapid pacing at 180 beats/min, achieving a mean invasive transvalvular gradient of 11 mm Hg (baseline 80 mm Hg) without paravalvular leak (Figure 5, Video 5). The Sapien 3 Ultra 29 valve was then implanted in the degenerated Biocor Epic 29 mitral prosthesis via right femoral vein access. After transseptal puncture with a SL0 sheath and dilator [St. Jude Medical], a guidewire was placed in the left superior pulmonary vein. With the support of an Agilis steerable catheter [Abbott], the mitral prosthesis was crossed using a pigtail catheter, through which 2 stiff Safari extra-small guidewires were advanced into the left ventricular apex. The atrial septostomy was performed with a 14-mm balloon, and the Sapien 3 Ultra 29 valve was implanted under rapid pacing at 160 beats/min (Figure 6, Video 6). Final TEE showed a mean mitral transvalvular gradient of 5 mm Hg with no paravalvular leak or LVOT obstruction (Video 7, Video 8).

During the procedure, midazolam 3 mg was administered in fractionated boluses, along with ketamine 120 mg aiming to maintain a Richmond Agitation-Sedation Scale score of −2/−3 and spontaneous breathing. To counterbalance the need for prolonged supine positioning, the patient was maintained on continuous positive airway pressure via a full-face mask. No alterations of vital or respiratory parameters were recorded. At the end of the procedure, the patient awakened promptly and was easily weaned from ventilatory support.

## Outcome and Follow-Up

In the following days, the patient showed progressive clinical improvement, leading to weaning from oxygen therapy and vasopressor support. He was discharged to a rehabilitation program in good condition after 6 days. Echocardiography performed at discharge showed the patient had a mean gradient of 23 mm Hg, an EOA of 1.2 cm^2^, a DVI of 0.35, and no leak at the level of the aortic bioprosthesis. The mitral valve showed a mean gradient of 8 mm Hg and no leak. At 4-year follow-up, the echocardiographic data remained largely stable, with a mean gradient of 25 mm Hg for the aortic prosthesis, an EOA of 1.0 cm^2^, and a DVI of 0.30. The mitral prosthesis at follow-up had a mean gradient of 8.5 mm Hg and no leak. At follow-up visits, the patient did not report any further exertional dyspnea and showed no signs of heart failure.

## Discussion

ViV implantation has emerged as the treatment of choice for degenerated surgical bioprostheses, particularly in patients at high risk for redo surgery.^6^ This case includes several unique aspects. First, it was not possible to perform a dedicated CT scan with arterial phase images for preprocedural evaluation of the degenerated surgical bioprostheses owing to the patient's critically unstable condition. However, using the images acquired in the venous phase from the CT scan initially performed in the ED to rule out PE, it was possible to measure the true ID of both prostheses and the aorto-mitral angle. The coronary obstruction risk was assessed using angiographic and TEE images. Another noteworthy aspect of this case is that both aortic and mitral ViV implantations were performed in a single session. Whereas this approach has been reported as feasible,^7^ in our scenario, it was a necessary choice owing to the patient's unstable condition. We decided to treat the aortic valve first to reduce afterload and optimize hemodynamics before proceeding with the mitral ViV. Implanting the mitral valve first could have reduced the available space in the LVOT, complicating subsequent aortic deployment and increasing the risk of valve malposition or displacement. A balloon-expandable valve was selected for the aortic ViV to minimize the risk of coronary obstruction, allow for quicker deployment, reduce the risk of LVOT impingement, and facilitate future transcatheter aortic valve replacement-in-transcatheter aortic valve replacement-in-surgical aortic valve replacement procedures in case of degeneration. Additionally, the procedure was performed under sedation with NIMV rather than standard general anesthesia, given the hemodynamic concerns.^8^ The immediate outcome of the procedure was good, especially from a clinical perspective, although the gradients at the follow-up were slightly elevated. However, questions arise about the long-term management of a young patient.

## Conclusions

Transcatheter aortic and mitral ViV implantation in the same procedure, performed under sedation with NIMV, appears to be a feasible and safe approach, even in patients in critically unstable condition. Furthermore, the 4-year follow-up indicates a satisfactory long-term outcome.

**Visual Summary:**
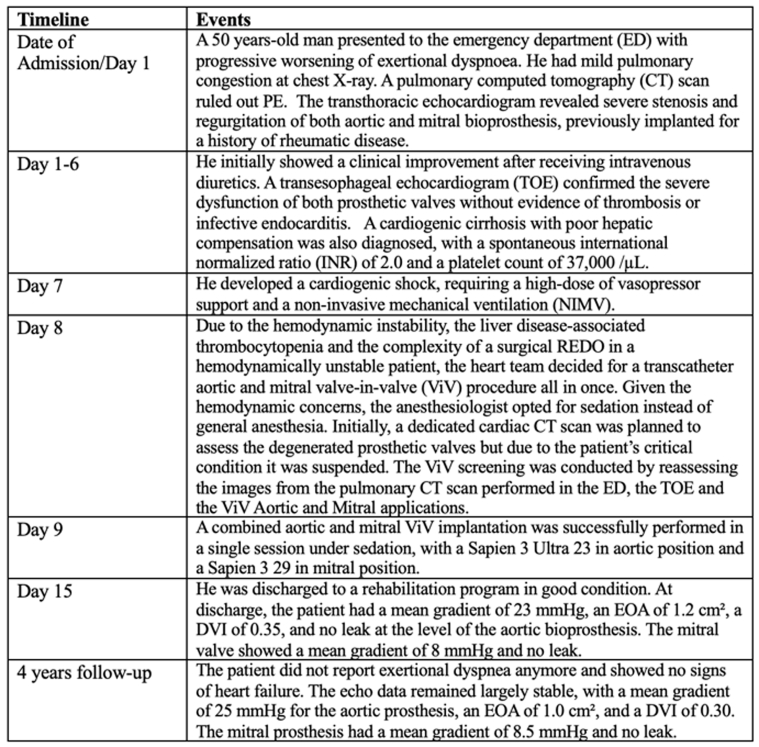
Timeline of the Case

## Funding Support and Author Disclosures

The authors have reported that they have no relationships relevant to the contents of this paper to disclose.

## Appendix

Video 1 Transthoracic Echocardiography, Apical View 5-Chamber View, Showing Aortic Bioprosthetic Valve Disfunction

Video 2 Transesophageal Echocardiography, 4-Chamber View, Showing Mitral Bioprosthetic Valve Disfunction

Video 3 Transesophageal Echocardiography, 4-Chamber View, Showing Mitral Bioprosthetic Valve Disfunction

Video 4 Angiography, Coplanar View Preintervention

Video 5 Angiography, Aortic Valve-in-Valve Postimplantation

Video 6 Intraprocedural Transesophageal Echocardiography, Mitral Valve-in-Valve Implantation

Video 7 Mitral Valve Postprocedural Transesophageal Echocardiography

Video 8 Mitral Valve Postprocedural Transesophageal Echocardiography

Video Summary Double Valve-in-Valve Under Sedation in Cardiogenic Shock A 50-year-old patient presented with severe bioprosthetic valve dysfunction and hemodynamic instability. Due to the patient's critical condition, standard ViV CT screening was not feasible. A transesophageal echocardiography, an emergency pulmonary CT scan and the ViV app were used to plan the intervention. A successful double ViV procedure was performed under deep sedation, avoiding general anesthesia. The patient was discharged in stable condition and remained event-free after 4 years.
